# Cell type-specific knock out models to unravel NO/cGMP signaling in smooth muscle

**DOI:** 10.1186/2050-6511-14-S1-O29

**Published:** 2013-08-29

**Authors:** Noomen Bettaga, Dieter Groneberg, Ronald Jäger, Barbara Lies, Andreas Friebe

**Affiliations:** 1 Physiologisches Institut, Universität Würzburg, Würzburg, Germany

## 

NO-sensitive guanylyl cyclase (NO-GC) is accepted to be the major receptor for the signaling molecule NO. Deletion of NO-GC in mice leads to disturbed NO/cGMP signaling. As a result, these mice show abolished NO-dependent relaxation of smooth muscle-containing tissues in the cardiovascular and gastrointestinal systems. Mice with general deletion suffer from increased blood pressure, reduced bleeding time, erectile dysfunction and die prematurely due to gastrointestinal dysmotility.

Several of the phenotypical changes caused by NO-GC deficiency are due to increased smooth muscle tone. Therefore, our work concentrated on NO/cGMP-mediated regulation of smooth muscle contraction/relaxation in various tissues. These include smooth muscle from blood vessels, gut, corpus cavernosum and lower urinary tract. NO-induced relaxation of all NO-GC-containing smooth muscle tissues was influenced by the deletion of the enzyme. To our surprise, the degree of smooth muscle dysfunction was not homogeneous: In contrast to vascular or urethral smooth muscle, gastric fundus and other GI muscles revealed an only a partially reduced NO-induced relaxation. Detrusor muscle of the bladder was unresponsive towards NO. Closer examination of the tissues showed variable expression of NO-GC in the different smooth muscle cells. Importantly, in addition to smooth muscle cells, many tissues showed NO-GC expression in other cell types such as endothelial cells, interstitial cells of Cajal and fibroblast-like cells.

We have generated various cell-specific KO strains using the inducible Cre-lox-system. In the GI tract we were able to show a dual mechanism of NO-induced relaxation via interstitial cell of Cajal and smooth muscle cells. In addition, we identified a third type of NO-GC-expressing cell, the fibroblast-like cell, whose function is still enigmatic. In penile corpus cavernosum, strong NO-GC expression was found in smooth muscle cells and, surprisingly, also in endothelial cells. The function of NO-GC in the endothelium is currently being investigated. Further studies using double or triple KO mutants will hopefully allow advising cell-specific functions of NO/cGMP signaling in murine smooth muscle.

